# Effect of pH on the In Vitro Biocompatibility of Surfactant-Assisted Synthesis and Hydrothermal Precipitation of Rod-Shaped Nano-Hydroxyapatite

**DOI:** 10.3390/polym13172994

**Published:** 2021-09-03

**Authors:** Dan-Jae Lin, Hao-Lian Lin, Ssu-Meng Haung, Shih-Ming Liu, Wen-Cheng Chen

**Affiliations:** 1 School of Dentistry, College of Dentistry, China Medical University, Taichung 404, Taiwan; djlin@mail.cmu.edu.tw; 2 The Ph.D. Program for Medical Engineering and Rehabilitation Science, China Medical University, Taichung 404, Taiwan; 3 Master Program for Biomedical Engineering, China Medical University, Taichung 404, Taiwan; 4 Biomaterials Translational Research Center, China Medical University Hospital, Taichung 404, Taiwan; 5 Advanced Medical Devices and Composites Laboratory, Department of Fiber and Composite Materials, Feng Chia University, Taichung 407, Taiwan; a50056a50056@gmail.com (H.-L.L.); dream161619192020@gmail.com (S.-M.H.); 0203home@gamil.com (S.-M.L.); 6 Department of Fragrance and Cosmetic Science, College of Pharmacy, Kaohsiung Medical University, Kaohsiung 807, Taiwan; 7 School of Dentistry, College of Dental Medicine, Kaohsiung Medical University, Kaohsiung 807, Taiwan

**Keywords:** hydroxyapatite, surfactant, template, biocompatibility, micelle, nanoparticles, in vitro

## Abstract

Given their wide range of biomedical applications, hydroxyapatite (HA) nanoparticles are an attractive material widely used in many fields. Therefore, a simple, inexpensive, and stable process for the synthesis of HA nanoparticles is necessary to meet current needs. Herein, we studied HA synthesis assisted by four surfactants, namely cation, anion, non-ionic, and zwitterion templates, to verify the synthesis phase, aspect ratio, morphology, and biocompatibility under different environments (i.e., pH 4 and 9) before and after calcination. Results showed that before calcination, the surfactant-free groups could not produce HA but showed an abundant dicalcium phosphate anhydrous (DCPA) phase at pH 4. Except for the anionic group containing a small amount of DCPA, all surfactant-assistant groups presented single-phase HA in acidic and alkaline environments. The diameter of HA synthesized at pH 4 was significantly larger than that of HA synthesized at pH 9, and the effect of aspect ratio changes after calcination was more significant than that before calcination. The uncalcined rod-shaped HA synthesized with a non-ionic template at pH 4 demonstrated excellent cell viability, whereas anionic, cationic, and non-ionic surfactants exhibited biocompatibility only after calcination. At pH 9, non-ionic and uncalcined zwitterion-assisted rod-shaped HA showed excellent biocompatibility. In conclusion, the uncalcined HA rod-shaped nanoparticles synthesized from the non-ionic template at pH 4 and 9 and the zwitterion template at pH 9, as well as all surfactant-assisted HA after calcination, had no cytotoxicity. These tailor-made non-toxic HA types can meet the different requirements of apatite composite materials in biomedical applications.

## 1. Introduction

Hydroxyapatite (HA) is the crystalline form of apatite. Almost all chemicals in the apatite family can promote bone growth by introducing bone-derived mechanisms. It does not cause local or systemic toxicity and foreign-body reactions [1,2]. As the average age of the world’s population increases, the need to repair or replace damaged and diseased tissues has increased significantly. When HA is implanted, it binds to bone tissue through chemical bonds, so it has good bone repairability. It is highly suitable as a repair material for bone defects. By providing the required surface and space for cell attachment, proliferation, migration, and differentiation to organize normal bone tissue, it is used as a scaffold for hard-tissue regeneration [3]. In addition, the controlled dissolution of bioceramic implants after contact with the biological solution provides the required ions for the three-dimensional (3D) micro-/nano-environment in the body. The main challenge in the synthesis of apatite powder is to precisely control the growth of HA crystals before manufacturing the required scaffolds for various tissues [3,4], which directly affects the size and geometry of the final nanoparticles [5]. The microscopic shape, size, and size distribution of HA can significantly affect the mechanical properties, processing conditions, surface chemistry, biocompatibility, and biological activity of HA [6,7].

Therefore, by controlling the size and shape of the HA crystal, the potential applications of HA can be expanded. For example, as a result of poor bending and tensile strength, HA bioceramics with random structures cannot be used in orthopedic applications that require load bearing [8,9]. Novel synthesis methods that precisely control the geometry of crystals must be developed. By adding an organic active agent to make HA crystallize into a suitable shape of HA, the growth of the HA crystal plane can be regular, forming spherical, flake/flaky, rod/whisker, and other HA types, which are scientifically feasible [10,11,12,13]. These types have led to different applications. For example, spherical HA is often used as a carrier to impregnate drugs, while plate-shaped HA is used to enhance the mechanical properties of composite materials. In particular, rod-shaped HA has a wide range of applications. It can be used as a carrier to absorb drug proteins or genes, and it can also be used as a carrier to effectively use its rod-shaped properties to improve the toughness of implant materials [14]. According to the literature, rod-shaped HA has good biocompatibility and biological activity because its shape is similar to that of human bone [15,16].

Hydrothermal synthesis is a simple chemical precipitation method for the synthesis of inorganic materials. Its advantage is that HA crystals with small volume and high purity can be prepared [17,18]. The reaction environment, temperature, and pH are the most important factors affecting the structure and morphological characteristics of HA particles. The pure phase cannot be easily synthesized under acidic environmental conditions, and most of it will be accompanied by the formation of other calcium salts and phosphates. Conversely, a stable HA phase can be prepared under alkaline conditions [18]. In addition, the biggest disadvantage of the hydrothermal method in producing HA nanorods is the inability to control the shape and size distribution. The particle shape obtained by the traditional hydrothermal method is usually irregular, spherical, or multi-rod; the particle size distribution is wide, which is the same as other chemical precipitates. To improve this phenomenon, scholars proposed the use of surfactants as template agents, which can effectively control the shape of HA particles [19].

Surfactant molecules are composed of two parts, which have different characteristics: the head group (hydrophilic) that has a strong affinity for solvents and the tail group (hydrophobic) that has a small affinity for solvents. Once dissolved, the head and tail groups can be negatively or positively charged. According to the literature, the ionic properties of surfactants can effectively control the shape of HA crystals during formation and lead to rods with different particle diameters and aspect ratios [20]. By changing the pH and different ratios of surfactants, the scale of the synthesized HA will also be changed [21,22]. According to previous studies, when the cationic surfactant concentration is greater than the critical micelle concentration (CMC) [23,24], the cationic surfactant (hexadecyltrimethylammonium bromide, CTAB) leads to a positive charge on the micelle surfaces in an aqueous solution. Given the electrostatic attraction between phosphate (PO_4_^3−^) ions and micelles, these negatively charged PO_4_^3−^ ions combine with the surface of positively charged micelles to form a network structure. The network slowly grows into rod-shaped micelles and becomes the nucleation center of HA. On the surface of the rod-shaped micelles, as the precipitation process proceeds, an increasing number of Ca^2+^ ions are adsorbed on the surface of the micelle/phosphate ion network, which can make HA crystals develop into a rod shape due to the repulsion of Ca^2+^ ions. The principles of anionic surfactants and amphoteric surfactants are the same. Although the micelles of non-ionic surfactants are not charged, their rod-shaped micelles are believed to attract HA crystals to follow rod-shaped micelles due to the polarization force [25].

Given that most HA prepared from anionic and cationic active agents is biologically toxic, most of the above studies require high-temperature calcination to remove toxicity. This study aimed to use different organic active surfactants, including anion, cation, non-ionic, and zwitterion templates. These four surfactants were processed into a hydrothermal reaction. A systematic comparison of the influence of HA morphology, structure, and biological activity before and after calcination of the organic surfactant template was conducted.

## 2. Materials and Methods

### 2.1. Raw Materials

Four different types of surfactants were selected in this study: cationic surfactant CTAB (Ferak Berlin GmbH, Berlin, Germany), anionic surfactant sodium dodecyl sulfate (SDS, purity > 99.0%), non-ionic surfactant Pluronic^®^-F127 (F127, *M_w_* = 12.6 kg mol^−1^), and zwitterionic surfactant cocamidopropyl betaine (CAPB). SDS, F127, and CAPB were all purchased from Sigma-Aldrich (St. Louis, MO, USA). Calcium hydrogen phosphate (CaHPO_4_·2H_2_O) was from HSE PureChem (Austin, TX, USA), and calcium hydroxide (Ca(OH)_2_) was from Shikamaru’s Pure Chemicals (Osaka, Japan).

### 2.2. Surfactant-Assisted HA Powder Synthesis

First, CaHPO_4_·2H_2_O and Ca(OH)_2_ powder with a calcium-to-phosphorus atomic ratio of 1.67 was added to 160 mL of deionized water and stirred well. Different surfactants were added to the solution. The addition amount was constant at 1 g, which confirmed that the micelles formed because the specified concentration was higher than the CMC. The pH of the solution was adjusted to 4 or 9 with 2 M acetic acid or sodium acetate aqueous solution. The solution was subjected to hydrothermal synthesis. The control group without surfactant-assisted synthesis was denoted as P, and the groups assisted with different surfactants were denoted as CTAB, SDS, F127, and CAPB. The reaction environment was 120 °C, and the temperature was maintained for 24 h. After the reaction, the solution was quickly aspirated and filtered to obtain the powder, washed with double-distilled water (ddH_2_O), and dried at 60 °C for 24 h as the prepared HA powder for the following comparison.

In addition, the surfactant was removed from the calcined group for comparison. The hydrothermally synthesized HA powder was sintered at a heating rate of 15 °C/min. After being heated to 900 °C, the temperature was maintained for 4 h and then cooled to room temperature to obtain calcined HA. The calcined control group was denoted as P-C, and the different surfactant-assisted synthesis groups were denoted as CTAB-C, SDS-C, F127-C, and CAPB-C.

### 2.3. Morphological Observation and Aspect Ratio

The shape and structure of the prepared HA were observed by scanning electron microscopy (SEM; S-3000N, Hitachi, Tokyo, Japan). Randomly selected HA powder (*n* = 30) was obtained to measure the length-to-diameter ratio under 10 k magnification.

The synthesized powder was also investigated using a field emission transmission electron microscope (FE-TEM, JEM-2100F; JEOL Co., Tokyo, Japan) equipped with an energy-dispersive X-ray spectrometer (EDS; X-MaxN TSR, OXFORD, Abingdon, England) and operated as a 200 kV field emission analytical electron microscope to identify the crystallinity. The samples were prepared by dispersing the powder in ethanol, collected through a 300-mesh copper mesh, and dried in an oven. Bright-field images, selective-area diffraction (SAD) patterns taken with an aperture size of 160 nm, or high-resolution lattice images were used for image and structural analyses. The software Digital Micrograph (Gatan) was used to measure the d-space of the diffraction plane.

### 2.4. X-ray Diffraction (XRD) Analysis

The powder was subjected to diffraction analysis by using an XRD diffractometer (XRD-6000; Shimadzu, Japan) with Ni-filtered Cu target Kα diffraction operated at 40 kV and 30 mA. The scanning speed was 2°/min in the scanning 2*θ* range of 20°–60°. Different phases were identified by comparing the diffraction peaks of the sample with the JCPDS standard file.

### 2.5. Fourier Transform Infrared Spectroscopy–Attenuated Total Reflection (FTIR-ATR)

The functional groups of the synthesized HA were analyzed by FTIR-ATR (Nicolet 6700, Thermo Fisher Scientific, Waltham, MA, USA). The HA powder and KBr were uniformly mixed in a ratio of 1:100 and pressed into a translucent disk with a diameter of 12 mm for FTIR-ATR analysis.

### 2.6. Biocompatible Evaluation

The mouse fibroblast cell line NIH-3T3 was cultured in Dulbecco’s modified Eagle medium (Gibco^®^, Invitrogen Taiwan Ltd., Taipei, Taiwan) containing 10% bovine serum (BS; Biolegend Co., San Diego, CA, USA) and 1% penicillin-streptomycin. The positive control used was 15 wt% dimethyl sulfoxide (DMSO; Sigma-Aldrich, Louis, MO, USA), which was sterilized by filtration through a 0.22 μm filter. High-density polyethylene was used as the negative control, and the weight-to-volume ratio (g/mL) of the extracted medium was 1/5. The control group used a normal cell culture medium to culture NIH-3T3 cells.

The preparation method of the extract of the experimental group was as follows: The powder was placed in the cell culture medium and extracted at 37 °C for 24 h in a ratio of 0.2 g/mL powder to culture medium. The medium was centrifuged at 3000 rpm for 5 min, and the supernatant was aspirated. About 100 μL of cells with a cell concentration of 1 × 10^4^ was obtained, and the sample extract (100 μL/well) was added to a 96-well plate for cell culture. After culturing for 24 h, the cell culture medium used in the experiment was aspirated, replaced with ordinary cell culture medium (100 μL/well), mixed with an XTT cell proliferation detection kit (50 μL/well; Sigma-Aldrich, Louis, MO, USA), and placed in a 37 °C, 5% CO_2_ incubator for 4 h. The viability of L929 cells was measured by absorbance at an optical density (OD) of 492 nm by using an ELISA plate reader (EZ Read 400; Biochrom, Cambridge, UK).

### 2.7. Statistical Analysis

The average aspect ratio of the HA crystals was analyzed by one-way ANOVA with post hoc tests, and the paired *t*-test was performed in IBM SPSS Statistics Version 20 (IBM, Armonk, NY, USA).

## 3. Results and Discussion

### 3.1. Characterization of the Hydrothermal Synthesis of HA by XRD

**Figure 1:**
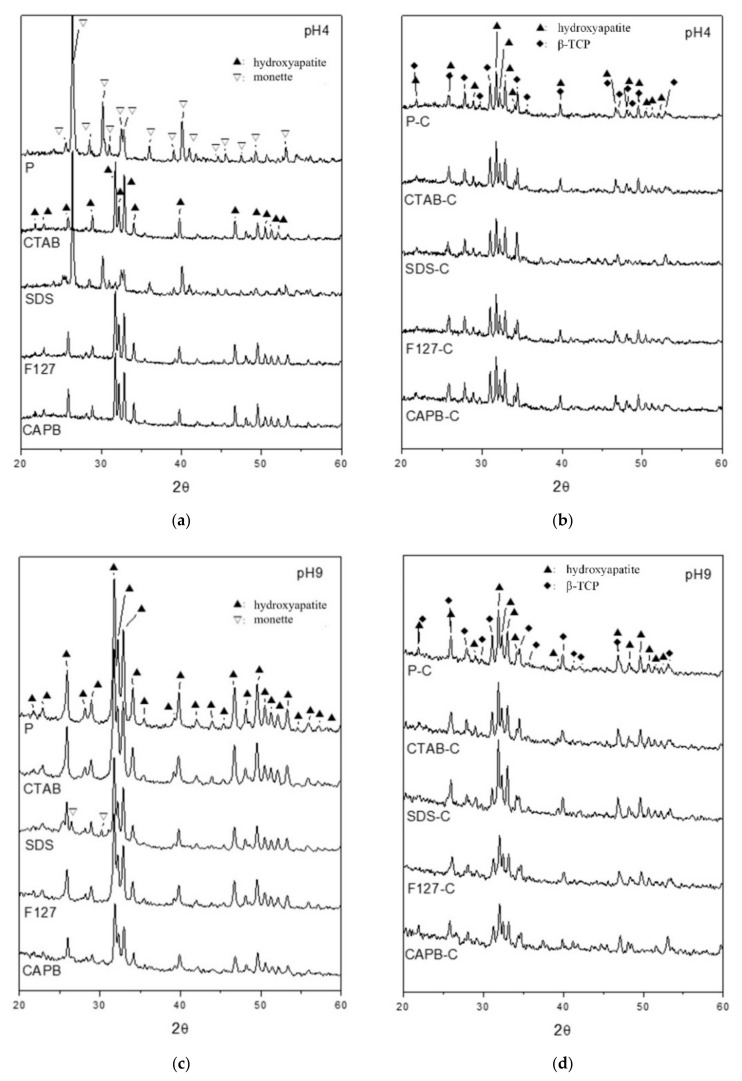
Comparison of XRD patterns by hydrothermal synthesis without a surfactant (P) and with the surfactant-assisted synthesis of CTAB, SDS, F127, and CAPB before and after calcination at 900 °C for 4 h (P-C, CTAB-C, SDS-C, F127-C, and CAPB-C) at (**a**) pH 4, (**b**) pH 4 after calcination, (**c**) pH 9, and (**d**) pH 9 after calcination.

The XRD comparison of the powder prepared by surfactant-assisted and hydrothermal preparation at 900 °C for 4 h under different environments of pH 4 and pH 9 is shown in Figure 1. In pH 4 hydrothermal synthesis (Figure 1a), the surfactant-free group (P) could not produce HA, and the main diffraction peaks at 2θ values of 26.4°, 30.2°, and 40.0° were assigned to corresponding (*hkl*) planes of (0 0 2), (−1 2 0), and (0 2 2), respectively, for the dicalcium phosphate anhydride (DCPA; CaHPO_4_, Monette: JCPDS 70-1425; 09-0080) phase. Under acidic conditions (pH < 4.8), the diffraction planes in the known DCPA structure were the same [26].

Notably, when using SDS as a nucleation template at pH 4, the diffraction peak was also based on the DCPA structure, although it contained a small amount of HA diffraction at 31.7°. When using a nucleating agent CTAB, F127, or CTAB as a nucleation template, HA planes of (2 1 1), (1 1 2), and (300) (HA: JCPDS 09-0432) had the highest diffraction density at 31.7°, 32.2°, and 32.9°, respectively, indicating that HA can be successfully synthesized even at pH 4 when surfactants CTAB, F127, and CAPB are used as nucleation templates. The diffraction patterns shown in Figure 1a,c revealed that the full-width at half-maximum (FWHM) of all HA diffractions at pH 4 (Figure 1a) was narrower than that at pH 9 (Figure 1c). The Scherrer equation *L = Kλ/(β*.cosθ) [27], which was developed in 1918, was used to calculate the nanocrystallite size (*L*) by XRD radiation of wavelength *λ* (nm) from measuring the FWHM of peaks (*β*) in radians, located at any 2θ in the pattern; the crystal degree increased as the FWHM decreased.

After calcination at 900 °C for 4 h (Figure 1b), the diffraction phases of all groups were similar. Except for the obvious HA diffraction peaks at 31.7°, 32.2°, and 32.9°, diffraction peaks at 31.0° and 34.4° corresponding to the (0 2 10) and (2 2 0) planes, respectively, for the beta-tricalcium phosphate (β-TCP: JCPDS 09-0169) phase in the rhombohedral structure were observed. Thus, the HA structure was partially degraded to β-TCP after calcination. When the powder was synthesized by the hydrothermal method in a pH 9 environment (Figure 1c), all the groups exhibited a single-phase HA structure, except for the SDS group containing a small amount of DCPA. After calcination in pH 9 synthesis (Figure 1d), all products contained biphasic HA and β-TCP, similar to powders calcined under pH 4 conditions. However, based on the relative height of β-TCP/HA diffractions, the β-TCP content in the pH 4 group was slightly higher than that in the pH 9 group.

HA has a hexagonal prism shape and produces hexagonal columnar crystals [28]. Therefore, the HA grown was calculated along the c-axis in the (002) crystal plane strength, and the (300) reflection was measured to evaluate the thickness of the HA along (100) [29]. The relative ratio was roughly reflected the changing trend by the aspect ratio. The XRD data are summarized in Table 1. The results showed that due to the β-TCP content in the pH 4 group being slightly higher than that in the pH 9 group, and considering the lattice structure of rhombohedral β-TCP as an extended stacking fault in hexagonal HA, the calcined energy led to a shift of several defects in the HA to a higher value. This led to the data obtained after the calcination of HA in the pH 4 group, as shown in Table 1, indicating that the ratio of the c-axis (002) plane to the thickness of HA (100) is not much different from the previous HA calcination. However, the HA of the pH 9 group showed that, except for the SDS group, the ratio of the HA growth of the c-axis (002) plane to the (100) thickness of each aspect ratio is significantly greater than that of the HA before calcination.

### 3.2. Characterization of HA by FTIR

**Figure 2:**
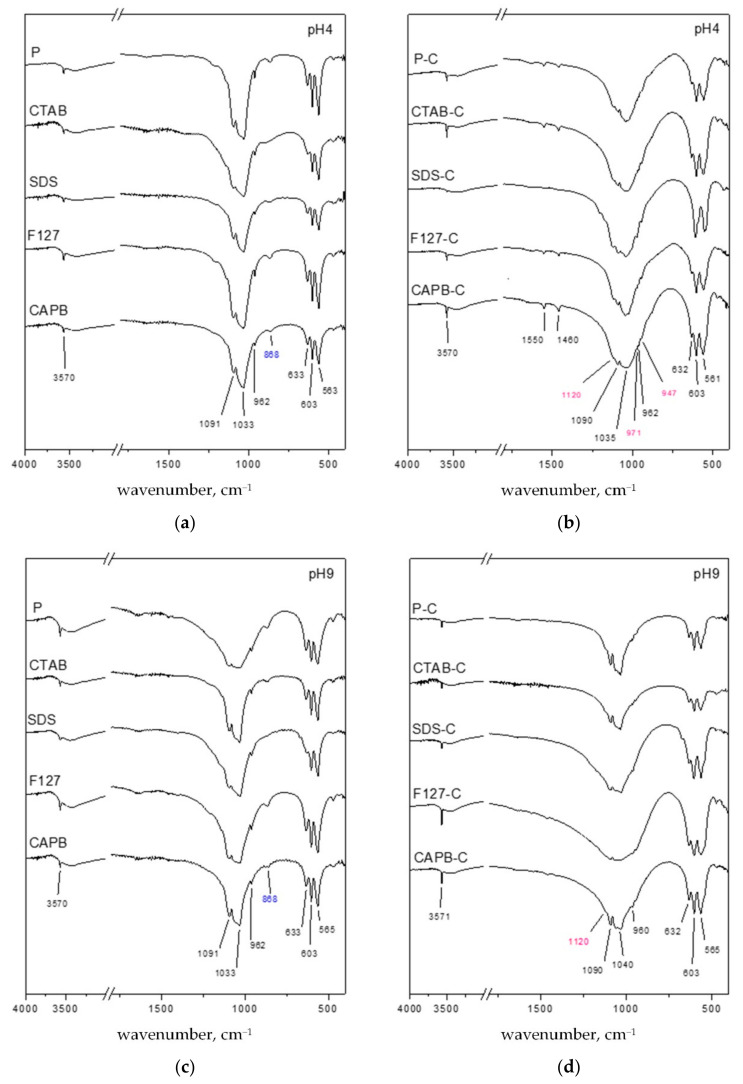
Comparison of FTIR spectra after hydrothermal synthesis without a surfactant (P) and assisted with four surfactants (CTAB, SDS, F127, and CAPB) after calcination at 900 °C for 4 h (P-C, CTAB-C, SDS-C, F127-C, and CAPB-C) at (**a**) pH 4, (**b**) pH 4 after calcination, (**c**) pH 9, and (**d**) pH 9 after calcination.

The FTIR results are shown in Figure 2. The absorption bands located at 3570 (ν_3_) and 633 cm^−1^ (ν_L_) were attributed to the asymmetric stretching and vibration modes of hydroxyl groups (OH^−^) in HA, respectively [30]. The absorption bands at 561–565 and 603 cm^−1^ were attributed to the harmonic bending vibration of the O–P–O bond (ν_4_ mode in PO_4_^3–^), and the absorption band at ~962 (ν_1_ mode in PO_4_^3–^) was due to the symmetrical stretching vibration [31]. The absorption bands at ~1033 and 1090 cm^−1^ were related to the asymmetrical stretching (ν_3_ mode in PO_4_^3–^) of phosphates of HA [30,31,32]. In Figure 2a, the band near 875 cm^−1^ may be due to P–O (H) stretching in the HPO_4_^2–^ groups, which was characteristic of DCPA or calcium-deficient apatite. When SDS used as a nucleation template at pH 4 is compared to the absorption bands of OH^−^ and PO (H) stretched in the HPO_4_^2−^ group, it shows that the HA and DCPA phases are consistent with the results obtained by XRD. The band at 3571 cm^−1^ corresponds to the vibration of the structure -OH group located along the c-axis in the HA crystal structure, and since P and SDS are templated at pH 4, it is a related low vertical sharp band. This may indicate that the inhibited fiber grows along the c -axis, which is consistent with the XRD results [33,34,35]. After HA was calcined (Figure 1b,d), β-TCP shoulder peaks began to appear, and the absorption bands at 947, 971, and 1120 cm^−1^ were assigned to the vibration mode of the PO_4_^3–^ group in β-TCP [36]. Compared with the HA synthesized at pH 9, the HA synthesized at pH 4 presented a clear β-TCP absorption band after calcination. This result was consistent with the β-TCP phase after calcination at pH 4 in XRD (Figure 1b).

For the HA synthesized at pH 4 after calcination, the IR absorption peak at 1460 cm^−1^ was assigned to the υ_3_ mode of the CO_3_^2–^ ions in apatite, which was typical of B-type CO_3_^2–^-containing apatite. Meanwhile, the peak at 1550 cm^−1^ belonged to the υ_3_ mode of A-type CO_3_^2–^ ions [37]. The FTIR spectrum showed that no adsorption bands were associated with organic molecules. Therefore, there was no residual surfactant in the product after calcination.

### 3.3. Morphology and Aspect Ratio

**Figure 3:**
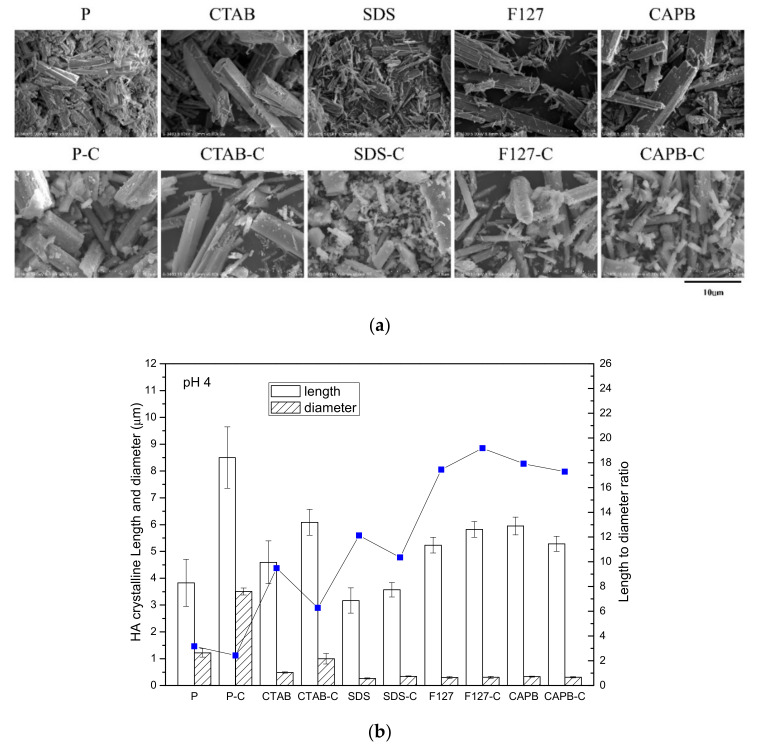
Powders before and after calcination in each group at pH 4. (**a**) SEM image and (**b**) particle size and the length-to-diameter ratio of the powder before and after calcination in each group.

Based on the SEM observations, at pH 4, in the surfactant-free group (P) (Figure 3a), the powder was plate and column shaped, with a large difference in size. In addition, after calcination (P-C group), the length doubled from ~4 μm to ~8 μm, and the diameter increased from ~1 μm to 3–4 μm, which showed that the crystals combined and coarsened after calcination.

**Figure 4:**
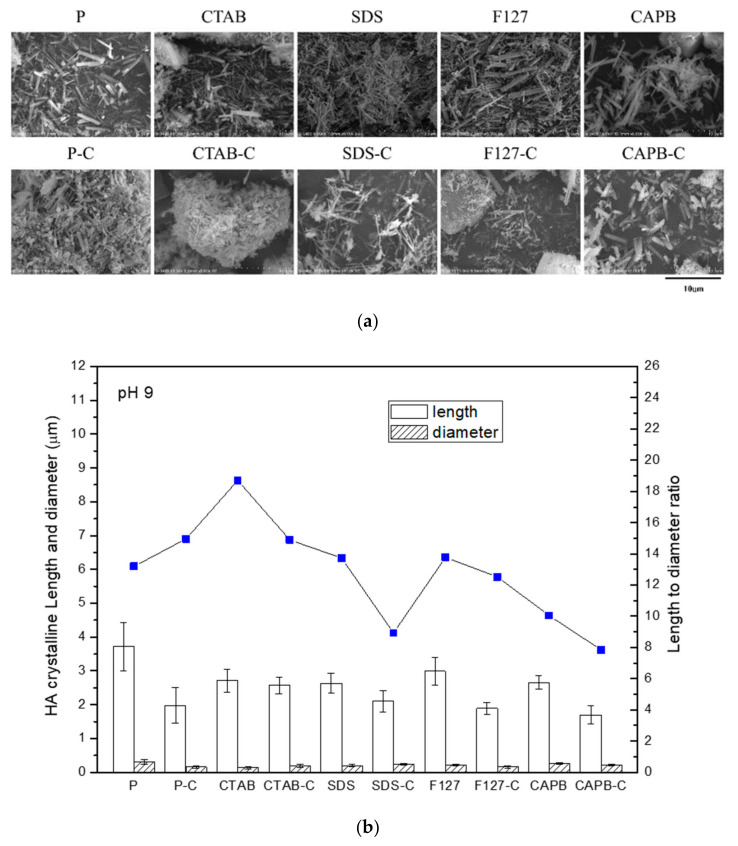
Powders before and after calcination in each group at pH 9. (**a**) SEM image and (**b**) particle size and the length-to-diameter ratio of the powder before and after calcination in each group.

In the group with interfacial surfactants, the morphology of the product powder was relatively close, which was evident from the large standard deviation of the length of the P and P-C groups in Figure 3b. In the CTAB group at pH 4, hexagonal columnar crystals with a length of about 5–10 μm and a diameter of about 2–3 μm were found to be HA crystals. The size of CTAB-C also increased slightly after calcination. The SDS surfactant template was the smallest HA crystal in pH 4 synthesis, with a length of only 3 μm and a diameter of only 200–300 nm. The aspect ratio of non-ionic (F127) and zwitterionic (CAPB) groups was ~18, which was much higher than that of the other groups, and the calcined aspect ratio and size changed slightly. These observations were related to dispersion after crystal growth. The more dispersed the crystal, the less affected it was by sintering. In general, the HA crystals synthesized at pH 9 were much smaller in size than those synthesized at pH 4 (Figure 4a). The length of the HA crystals was only 2–3 μm, and the diameter was about 150–250 nm. Except for the cationic CTAB group, the length of these crystals hardly changed after calcination. The crystals of the other groups (P, SDS, F127, and CAPB) were significantly shorter in length after calcination. The length-to-diameter ratio of each group showed a significant downward trend, and the P group was significantly shortened after calcination (Figure 4b). Although most of the trends obtained from the two results in Figure 4 and Table 1 are consistent, the difference between the measured crystal aspect ratio (Figure 4) and the one calculated from the XRD diffraction patterns (Table 1) is due to the phasic combination of HA and DCPA or β-TCP.

### 3.4. TEM Analysis

**Figure 5:**
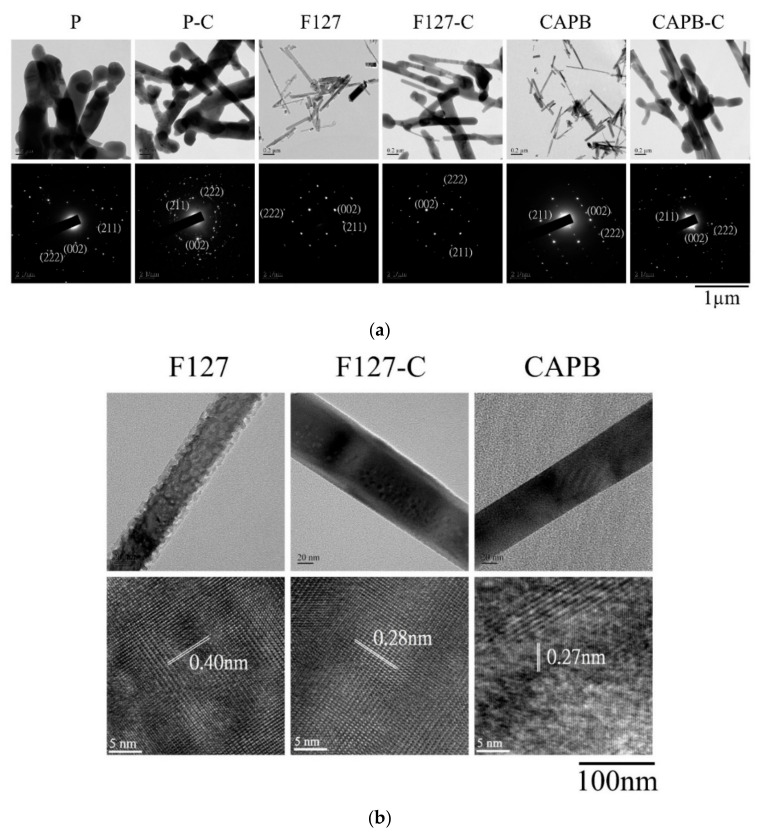
HA synthesized under pH 9 conditions, without adding surfactants (P, P-C) and with the surfactant-assisted synthesis of non-ionic surfactant F127 (F127, F127-C) and zwitterionic surfactant CAPB (CAPB, CAPB-C) to form rod-shaped HA crystals. (**a**) Upper row: bright-field image; lower row: selective-area diffraction pattern indexed with HA lattice-reflective planes. (**b**) Upper row: high-magnification bright-field image; lower row: lattice image of the rod HA crystal.

Figure 5a shows the comparison of TEM bright-field and SAD images between rod-shaped HA crystals and the control group before and after calcination with F127 and CAPB at pH 9. The rod-shaped HA crystals synthesized with F127 and CAPB as templates were nanometer-size in diameter and thickened after calcination. The crystals of the control group without a surfactant (P) were irregular and inconsistent in size. In the SAD results, the image showed obvious HA diffraction positions of the (0 0 2), (2 1 1), and (2 2 2) planes. The small crystals in the F127 and CAPB groups were chosen to facilitate HA to observe the lattice image. In Figure 5b, 0.40 nm corresponded to the (200) plane HA crystal, 0.28 nm corresponded to the (2 1 1) plane, and 0.27 nm corresponded to the (300) plane. Therefore, the XRD analysis results were confirmed.

### 3.5. Biocompatibility

**Figure 6:**
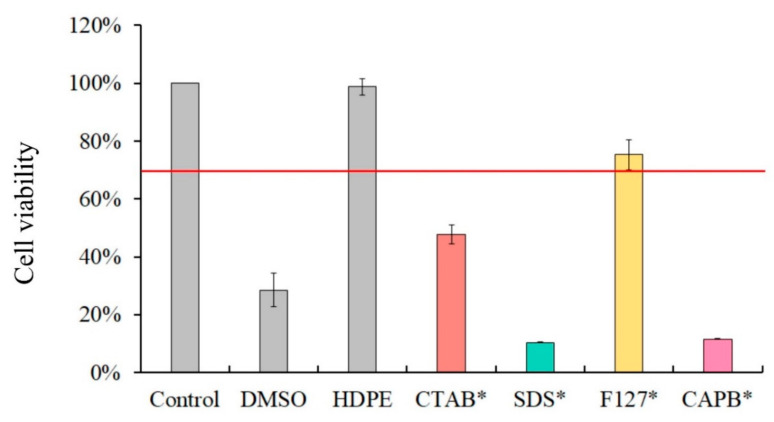
Cytotoxicity results of four surfactants only: CTAB *-, SDS *-, F127 *-, and CAPB *-cultured NIH-3T3 cells for 24 h.

The results of the cytotoxicity comparison of the four surfactants (CTAB, SDS, F127, and CAPB; Figure 6) demonstrated that non-ionic F127 has the lowest toxicity at the same concentration and exhibits more than 70% cell viability. The surfactants SDS and CAPB caused the highest cytotoxicity, and the cell viability was even lower than that of the positive control group DMSO.

**Figure 7:**
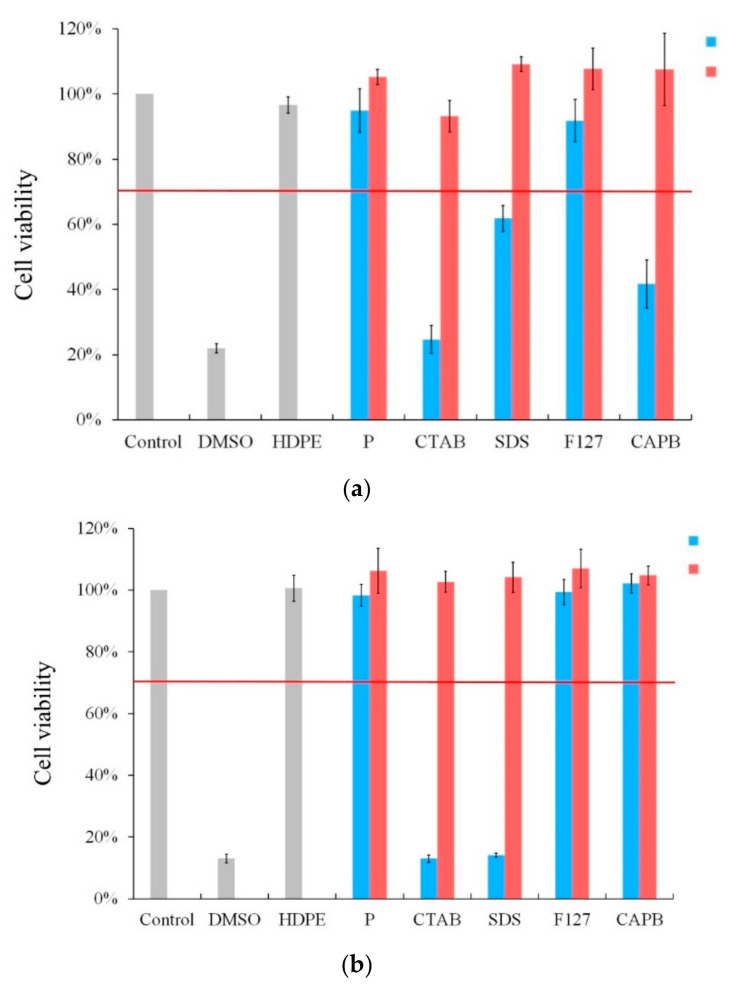
Cytotoxicity test of HA synthesized at (**a**) pH 4 and (**b**) pH 9 in hydrothermal synthesis without a surfactant (P) and with different surfactants: CTAB, SDS, F127, and CAPB (column before calcination: blue; column after calcination: red).

The cytotoxicity test was performed on HA crystals synthesized by the four surfactants under pH 4 conditions (Figure 7a). The uncalcined F127 group had excellent biocompatibility with the surfactant-free P group. However, in the surfactant-assisted CTAB, SDS, and CAPB uncalcined HA groups, cell viability dropped to less than 70% after 24 h of culture, showing cytotoxicity. After calcination at 900 °C, there was no cytotoxicity in each group.

When HA was synthesized at pH 9, the uncalcined rod-shaped HA crystals of the non-ionic F127 and CAPB groups maintained cell viability after 24 h of culture, but cell death was obvious in the CTAB and SDS groups. Similar to the results of pH 4, each group demonstrated excellent biological activity and no cytotoxicity after calcination at 900 °C.

## 4. Conclusions

Rod-shaped HA with good dispersibility and purity may be synthesized by the hydrothermal method and with a suitable interface surfactant template. It has potential for many biomedical applications, such as the manufacture and use of 3D-printed scaffolds, HA and polymer-based composite materials, and a carrier for drug delivery. Rod-shaped HA types all show superior properties, including biocompatibility and different aspect ratios, customized sizes, active surfaces to facilitate the grafting of biomolecules, and easy manufacturing, modification, and delivery applications. Previous studies have usually required precise pH, temperature control, or calcination to obtain pure rod-shaped HA. This study used non-ionic F127 or zwitterionic CAPB as a template. When the concentration of the organic active agent was greater than the CMC, highly dispersed, pure, rod-shaped HA crystals were obtained at pH 4 and pH 9. The HA synthesized under the pH 4 environment was coarse, while the HA synthesized at pH 9 was fine. Using non-ionic F127 as a template, the rod-shaped HA crystals synthesized hydrothermally at pH 4 exhibited excellent cell viability. In contrast, although FTIR confirmed that there was no residual surfactant, the rod-shaped HA crystals synthesized by the CTAB, SDS, and CAPB groups had excellent biocompatibility only after calcination at 900 °C. At pH 9, the rod-shaped HA crystals of the non-ionic F127 and zwitterionic CAPB groups without calcination showed excellent biocompatibility. To meet the current needs in the biomedical field, non-toxic HA nanoparticles have been synthesized and are expected to be used in various biomedical applications.

## Figures and Tables

**Table 1:** Summary of the XRD diffraction phase structure of hydrothermally synthesized calcium phosphates before and after calcination at 900 °C for 4 h at pH 4 and pH 9.

Surfactants			Synthetic Product Phases and Length-to-Thickness Ratio					
	pH 4				pH 9			
	Product Orginal Phase	Phase after Calcination	The c-axis (002) Plane/(100) Thickness of HA Aspect Ratio		Product Orginal Phase	Phase after Calcination	The c-axis (002) Plane/(100]) Thickness of HA Aspect Ratio	
			Original	Calcined			Original	Calcined
Surfactant-free P	DCPA	HA + β-TCP	0.50	0.68	HA	HA + β-TCP	0.63	0.90
Cationic, CTAB	HA	HA + β-TCP	1.03	0.80	HA	HA + β-TCP	0.74	0.87
Anionic, SDS	DCPA + (HA) ^a^	HA + β-TCP	0.63	0.53	HA + (DCPA) ^a^	HA + β-TCP	0.82	0.78
Non-ionic, F127	HA	HA + β-TCP	0.68	0.89	HA	HA + β-TCP	0.63	1.19
Zwitterionic, CAPB	HA	HA + β-TCP	0.72	0.72	HA	HA + β-TCP	0.81	1.68

^a^ Parentheses indicate the minor phase of the product.

## Data Availability

Data are contained within the article.
